# Impact of the COVID-19 pandemic and initial period of lockdown on the mental health and well-being of adults in the UK

**DOI:** 10.1192/bjo.2020.79

**Published:** 2020-08-17

**Authors:** Ross G. White, Catharina Van Der Boor

**Affiliations:** Institute of Population Health, University of Liverpool, UK; Institute of Population Health, University of Liverpool, UK

**Keywords:** Anxiety disorders, depressive disorders, coronavirus, COVID-19, pandemics

## Abstract

The impact of the COVID-19 pandemic on mental health and well-being were assessed in a convenience sample of 600 UK adults, using a cross-sectional design. Recruited over 2 weeks during the initial phase of lockdown, participants completed an online survey that included COVID-19-related questions, the Hospital Anxiety and Depression Scale, the World Health Organization (Five) Well-Being Index and the Oxford Capabilities Questionnaire for Mental Health. Self-isolating before lockdown, increased feelings of isolation since lockdown and having COVID-19-related livelihood concerns were associated with poorer mental health, well-being and quality of life. Perceiving increased kindness, community connectedness and being an essential worker were associated with better mental health and well-being outcomes.

On 24 March 2020, the UK introduced a range of ‘lockdown’ restrictions intended to slow the progression of the COVID-19 outbreak. Emerging evidence indicates that the COVID-19 pandemic is associated with adverse mental health outcomes for healthcare workers in China.^1^ Specifically, being female and having an intermediate level of seniority were associated with experiencing severe depression, anxiety and distress. A study conducted with the general population in Italy indicated that COVID-19-related stressful life events were associated with increased odds of post-traumatic stress, depression, anxiety, insomnia, perceived stress and adjustment disorder symptoms.^2^ There is, however, limited data relating to the potential impact of the COVID-19 pandemic on levels of well-being and quality of life (QoL). The capability approach,^3^ which focuses specifically on the extent to which people have the freedom to engage in valued forms of being and doing, provides a potentially important framework for understanding the impact of the COVID-19 pandemic and the associated lockdown. The lockdown restrictions associated with the pandemic have posed an inherent risk of isolation and a reduction in social connectedness. Research has highlighted that social connections can have positive effects on health and well-being.^4^ The current study, which is part of a programme of research aimed at tracking the impact of the COVID-19 outbreak, investigated whether mental health, well-being and QoL outcomes in UK adults are associated with experiencing symptoms of COVID-19, being in a group vulnerable to COVID-19 (the question read ‘I am classified as being in a vulnerable group in terms of COVID-19 (aged 70 or above, heart disease, lung disease, pregnant, etc)’), being categorised as an ‘essential worker’, experiencing COVID-19-related isolation and local community interactions. Further, the study explored if participants' level of social support was associated with mental health and well-being outcomes.

## Method

The authors assert that all procedures contributing to this work comply with the ethical standards of the relevant national and institutional committees on human experimentation and with the Helsinki Declaration of 1975, as revised in 2008. All procedures involving human participants were approved by the Central University Research Ethics Committees, University of Liverpool (reference 7633). Written informed consent was obtained from all participants.

A cross-sectional design was used. A convenience sample recruited via social media forums (Twitter, Facebook, Reddit) completed an online survey. Data was collected over 2 weeks in the initial lockdown period (31 March to 13 April 2020). To be eligible, people had to be adults (≥18 years), speak English and be living in the UK at the time of the COVID-19 outbreak.

The survey included demographic questions; COVID-19-related questions; the Hospital Anxiety and Depression Scale^5^ (HADS; higher scores on the subscales indicate higher levels of depression and anxiety symptoms); the World Health Organization (Five) Well-Being Index (WHO-5),^6^ a measure of well-being (higher scores indicate higher levels of well-being); the Oxford Capabilities Questionnaire for Mental Health (OXCAP-MH),^7^ a measure of QoL (higher scores indicate higher levels of QoL) and the Multidimensional Scale of Perceived Social Support^8^ (higher scores indicate higher levels of perceived social support).

A total of 600 participants (74% female, mean age 36.75 years, s.d. 13.44, range 18–76 years) completed at a minimum the demographic and COVID-19-related questions. Participants were mainly White (93.6%) and employed (65%). Around a quarter of participants (26.3%) self-reported currently receiving treatment for mental disorders, including mood disorders (18%) and neurotic, stress-related and somatoform disorders (14.3%). No participants had been diagnosed with COVID-19.

## Results

The mean scores on the HADS Anxiety subscale (mean 10.23, s.d. 4.98) and HADS Depression subscale (mean 7.57, s.d. 4.39) exceeded the normal range (i.e. scores of 0–7). The mean scores on the WHO-5 and OXCAP-MH were 10.43 (s.d. 5.40) and 69.45 (s.d. 11.91), respectively. Female participants reported significantly higher levels of anxiety symptoms (*t*(195.73) = −2.21, *P* = 0.028) than males (female mean 10.51, s.d. 4.85; male mean 9.33, s.d. 5.29). There were no significant differences in the level of depression symptoms, well-being and QoL between males and females.

Being in a vulnerable group (12.5%) or experiencing symptoms of COVID-19 (11.7%) were not associated with significant differences in mental health and well-being outcomes (see Table 1). Participants who self-isolated before lockdown owing to symptoms of COVID-19 (11.8%) had higher levels of anxiety (*t*(584) = 2.77, *P* = 0.006) and depression (*t*(550) = 2.83, *P* = 0.005) symptoms, and lower levels of well-being (*t*(534) = −2.29, *P* = 0.022) and QoL (*t*(466)= −3.56, *P* < 0.001), relative to those who did not. Participants who felt more isolated than usual during lockdown (69%) had higher levels of anxiety (*t*(513) = −5.95, *P* < 0.001) and depression (*t*(250.86)= −7.77, *P* < 0.001) symptoms, and lower levels of wellbeing (*t*(191.84) = 6.18, *P* < 0.001) and QoL (*t*(441) = 4.16, *P* < 0.001).

**Table 1:** Between-group analyses for HADS, OXCAP-MH and WHO-5

Question	Response	Mean	s.d.	*t*-value	d.f.	*P-*value
Being in a ‘vulnerable group’						
HADS Depression	Yes	7.80	4.65	0.48	549	0.629
	No	7.53	4.36			
HADS Anxiety	Yes	9.81	5.15	−0.75	546	0.454
	No	10.29	4.97			
OXCAP-MH	Yes	68.73	13.20	−0.63	465	0.527
	No	69.71	11.59			
WHO-5	Yes	10.44	5.83	0.04	532	0.970
	No	10.41	5.34			
Experienced symptoms of COVID-19						
HADS Depression	Yes	7.68	3.40	0.22	546	0.825
	No	7.55	4.46			
HADS Anxiety	Yes	10.56	4.74	0.56	544	0.573
	No	10.19	5.01			
OXCAP-MH	Yes	66.78	14.64	−1.60	65.72	0.113
	No	70.02	11.32			
WHO-5	Yes	9.82	5.46	−0.99	530	0.322
	No	10.52	5.40			
Self-isolated before lockdown owing to symptoms of COVID-19						
HADS Depression	Yes	9.00	4.36	2.83	550	0.005**
	No	7.37	4.36			
HADS Anxiety	Yes	11.83	4.74	2.77	548	0.006**
	No	10.02	4.98			
OXCAP-MH	Yes	64.42	13.25	−3.56	466	<0.001***
	No	70.28	11.43			
WHO-5	Yes	8.98	5.29	−2.29	534	0.022*
	No	10.63	5.39			
Agree that they felt more isolated than usual during lockdown						
HADS Depression	Yes	8.20	4.31	−7.77	250.86	<0.001***
	No	5.16	3.68			
HADS Anxiety	Yes	10.91	4.69	−5.95	513	<0.001***
	No	7.99	5.14			
OXCAP-MH	Yes	68.53	11.46	4.16	441	<0.001***
	No	73.67	11.00			
WHO-5	Yes	9.64	5.07	6.18	191.84	<0.001***
	No	13.17	5.67			
Identified as an essential worker						
HADS Depression	Yes	7.01	4.04	−2.18	400.76	0.030*
	No	7.84	4.54			
HADS Anxiety	Yes	9.83	4.79	−1.30	546	0.194
	No	10.42	5.08			
OXCAP-MH	Yes	70.30	11.40	0.97	465	0.332
	No	69.18	12.02			
WHO-5	Yes	10.82	5.12	1.18	532	0.238
	No	10.24	5.54			
Agree that the COVID-19 outbreak was threatening their livelihood						
HADS Depression	Yes	8.08	4.49	−2.55	544	0.011*
	No	7.13	4.23			
HADS Anxiety	Yes	10.67	4.93	−1.90	542	0.058
	No	9.86	4.96			
OXCAP-MH	Yes	68.05	11.92	2.73	461	0.007**
	No	71.02	11.47			
WHO-5	Yes	10.20	5.39	0.86	528	0.391
	No	10.61	5.39			
Agree that people's kindness toward others in their local area had increased						
HADS Depression	Yes	7.29	4.22	2.25	551	0.025*
	No	8.20	4.70			
HADS Anxiety	Yes	10.13	4.84	0.75	548	0.455
	No	10.47	5.29			
OXCAP-MH	Yes	71.09	11.12	−4.56	467	<0.001***
	No	65.74	12.75			
WHO-5	Yes	10.85	5.24	−2.85	535	0.005**
	No	9.42	5.62			
Agree that since the COVID-19 outbreak commenced they felt more connected to the members of their local community						
HADS Depression	Yes	7.07	4.08	2.11	552	0.035*
	No	7.87	4.56			
HADS Anxiety	Yes	10.00	4.87	0.85	549	0.395
	No	10.37	5.05			
OXCAP-MH	Yes	72.00	10.02	−3.87	467	<0.001***
	No	67.76	12.74			
WHO-5	Yes	11.24	5.06	−2.83	536	0.005**
	No	9.90	5.55			

HADS, Hospital Anxiety and Depression Scale; OXCAP-MH, Oxford Capabilities Questionnaire for Mental Health; WHO-5, World Health Organization (Five) Well-Being Index.

*P < 0.05; **P < 0.01; ***P < 0.001.

Participants who were essential workers (32%) had significantly lower levels of depression symptoms (*t*(400.76) = −2.18, *P* = 0.030). Participants who agreed that the COVID-19 outbreak was threatening their livelihood (46.0%) had higher levels of depression symptoms (*t*(544) = −2.55, *P* = 0.011) and lower QoL (*t*(461) = 2.73, *P* = 0.007).

Participants who agreed that people's kindness toward others in their local area had increased since the COVID-19 outbreak (68.8%) had lower levels of depression symptoms (*t*(551) = 2.25, *P* = 0.025), and higher QoL (*t*(467) = −4.56, *P* < 0.001) and well-being (*t*(535) = −2.85, *P* = 0.005). Similarly, participants who agreed that they had felt more connected to the members of their local community since the COVID-19 outbreak (40.0%) had lower levels of depression symptoms (*t*(552) = 2.11, *P* = 0.035), and higher QoL (*t*(467) = −3.87, *P* < 0.001) and well-being (*t*(536) = −2.83, *P* = 0.005).

The level of perceived social support had significant negative correlations with levels of depression (*r* = −0.33, *P* < 0.001) and anxiety (*r* = −0.17, *P* < 0.001) symptoms, and significant positive correlations with QoL (*r* = 0.52, *P* < 0.001) and well-being (*r* = 0.29, *P* < 0.001).

## Discussion

This study sought to investigate the impact of the COVID-19 outbreak on the mental health and well-being of a convenience sample of UK adults. The levels of anxiety and depression symptoms for the sample were markedly higher than normative data derived for the UK adult population's levels of anxiety (females 6.78, s.d. 4.23; males 5.51, s.d. 4.04) and depression (females 4.12, s.d. 3.78; males 3.83, s.d. 3.74)^9^ symptoms.

Higher levels of depression symptoms were associated with participants having to self-isolate before lockdown owing to symptoms of COVID-19, feeling more isolated than usual during lockdown or agreeing that the COVID-19 pandemic was threatening their livelihood. On the other hand, agreeing that people's kindness toward others had increased, agreeing that they felt more connected to people in the local community, and working in an essential job were associated with significantly lower levels of depression symptoms. Notably, the mean depression score for the essential workers (mean 7.01, s.d. 4.04) remained at the upper limit of the normal range. These findings are open to interpretation, but it may be that the importance of their work and/or public acknowledgment of their efforts buffered against higher levels of depression symptoms.

The significant findings relating to isolation (self-isolating before lockdown or feeling more isolated during lockdown) and levels of perceived social support highlight the importance of exploring innovative ways to maintain connection and social support during periods of lockdown and beyond. These findings, although correlational in nature, are consistent with the thesis that psychological resources associated with social connectedness can serve as a ‘social cure’ for mental health difficulties.^10^

Comparatively high levels of both well-being and QoL were associated with participants agreeing that levels of kindness in the local area had increased, and that they felt more connected to others in the local community during the COVID-19 pandemic. QoL, but not well-being, was comparatively lower in participants who indicated that their livelihood was threatened by the COVID-19 pandemic. We propose that the OXCAP-MH, as a multidimensional measure of QoL that incorporates a focus on a range of factors including non-health issues and welfare inequalities, is a valuable measure for assessing how COVID-19 and related restrictions are potentially affecting people.

There were a number of important limitations associated with the current study. The convenience sample relied on people who had access to online social media forums. Consistent with other studies that have used social media for recruitment,^11^ males and Black, Asian and minority ethnic community members were comparatively under-represented in the sample. The cross-sectional nature of the analyses limits the conclusions that can be drawn. However, forthcoming academic papers from the authors will track the impact of the COVID-19 pandemic and lockdown restrictions on mental health and well-being over time.

The study highlights that although there was no association between personal experience of COVID-19 symptoms and being part of a group vulnerable to the effects of COVID-19, and mental health and well-being, factors related to isolation and COVID-19-related livelihood concerns were in fact associated with poorer mental health and well-being. On the other hand, perceiving increased kindness and connectedness in local areas were associated with better mental health and well-being outcomes. Further research aimed at mitigating the mental health and well-being effects of public health emergencies is required.

## Data Availability

The data that support the findings of this study are available from the corresponding author upon reasonable request.
